# In Vivo Models to Study the Pathogenesis of Extra-Respiratory Complications of Influenza A Virus Infection

**DOI:** 10.3390/v13050848

**Published:** 2021-05-06

**Authors:** Edwin Veldhuis Kroeze, Lisa Bauer, Valentina Caliendo, Debby van Riel

**Affiliations:** Department of Viroscience, Erasmus University Medical Center Rotterdam, 3015 CN Rotterdam, The Netherlands; edwinvk@gmail.com (E.V.K.); l.bauer@erasmusmc.nl (L.B.); v.caliendo@erasmusmc.nl (V.C.)

**Keywords:** influenza, mammalian animal models, extra-respiratory disease, cardiovascular disease, central nervous system disease, pathogenesis, olfactory nerve, cranial nerves

## Abstract

Animal models are an inimitable method to study the systemic pathogenesis of virus-induced disease. Extra-respiratory complications of influenza A virus infections are not extensively studied even though they are often associated with severe disease and mortality. Here we review and recommend mammalian animal models that can be used to study extra-respiratory complications of the central nervous system and cardiovascular system as well as involvement of the eye, placenta, fetus, lacteal gland, liver, pancreas, intestinal tract, and lymphoid tissues during influenza A virus infections.

## 1. Introduction

Even though influenza A viruses (IAVs) may cause mild upper respiratory tract (RT) disease, in severe cases, extra-respiratory complications occur frequently. The most common extra-respiratory complications involve the central nervous system (CNS) and cardiovascular system [1]. Since the pathogenesis of these, often severe, complications are poorly understood, relevant animal models are an important tool to study the systemic pathogenesis as well as efficacies of vaccines or antivirals. In this review, we elaborate on established in vivo models to study the extra-respiratory spread of IAVs and the pathogenesis of the associated complications.

Animal models to study the pathogenesis of influenza encompass more than the animal species alone; it also includes the route of virus inoculation as well as the virus subtype and infectious dose. The immune status of the host is also an important factor in the pathogenesis of influenza, which can be altered due to the health status (e.g., obesity or pregnancy), or due to medication. The experimental animal models described here include the laboratory mouse and domestic ferret, but also models that are less commonly used, such as non-human primates, cats, red foxes, and hamsters. An important inclusion criterion for this review was that the model shows IAV-induced extra-respiratory disease in conjunction with detectable virus infection (by virus isolation) and/or replication (by immunohistochemistry). Since extra-respiratory spread of influenza A viruses is rarely observed in guinea pigs and pigs after experimental inoculation with influenza A virus, these species are not included in this review.

Extra-respiratory spread and associated complications in mammals including humans are most commonly observed following infection with highly pathogenic avian influenza viruses (HPAIVs). This contrasts to seasonal human IAVs such as subtypes H3N2 and H1N1 that are, in general, confined to replication in the RT of humans. Pandemic IAVs, such as 2009 pandemic H1N1 virus (2009 pH1N1 virus) and reconstructed pandemic 1918 H1N1 virus, usually have an intermediate phenotype in extra-respiratory spread and associated inflammation. Many factors contribute to the ability of IAVs to spread beyond the RT, such as the presence of a multibasic cleavage site in the hemagglutinin of IAV, the ability to attach to host cells, and the immune status of the host [2,3]. However, these factors will not be discussed in this review.

We organized this review in chapters according to the organ system involved: central nervous system (CNS) disease, cardiovascular disease, and disease that involves other organ systems. This inevitably introduces some overlaps between the chapters that describe the same studies from literature reporting on various organ systems. Throughout this review, the term ‘inoculation’ is used to denote specifically experimental IAV inoculation, whilst ‘infection’ is reserved for virus infection of cells following virus inoculation or transmission. Various methods and routes of virus inoculation are discussed throughout this review. Common inoculation methods are abbreviated throughout as follows: intratracheal (i.t.), intranasal (i.n.), intra-gastro-intestinal (g.i.), and intraocular (i.o.). Furthermore, within this review, the term ‘animal model’ is meant to denote the complete set of variables that typify the model. The experimental animal species described include: the laboratory mouse (*Mus musculus domesticus*), domestic ferret (*Mustela putorius furo*), red fox (*Vulpes vulpes*), domestic cat (*Felis catus*), Syrian hamster (*Mesocricetus auratus*), and nonhuman primates (NHPs) such as the cynomolgus macaque (*Macaca fascicularis*), rhesus macaque (*Macaca mulatta*), common marmoset (*Callithrix jacchus)*, and the African green monkey or vervet monkey (*Chlorocebus aethiops*).

## 2. CNS Disease

In humans, the most common extra-respiratory complications of influenza are linked to the central nervous system (CNS). IAV infections may incite acute CNS disease including encephal(omyel)itis, meningitis, febrile seizures, and stroke, and, less frequently, chronic CNS diseases such as Guillain-Barré syndrome [4,5], Reye’s syndrome (reviewed in [1]), encephalopathy [6,7], post-encephalitis parkinsonism [8], and transverse myelitis [9,10]. Acute disease is most frequently observed after infections with zoonotic HPAIVs followed by infections with pandemic IAVs (1918 H1N1 virus or 2009 pH1N1 virus) and seasonal IAVs [11,12]. In this section, we will focus on established animal models to study acute IAV-induced CNS disease.

Virus spread to the CNS may occur directly from the RT, via cranial nerves that innervate the RT, or via hematogenous spread and subsequent spread across the blood-brain-barrier or blood-cerebrospinal fluid (CSF)-barrier. Thus far, evidence for virus spread to the CNS via cranial nerves I (Olfactory nerve), V (Trigeminal nerve), X (Vagus nerve), VII (Facial nerve), VIII (Vestibulocochlear nerve), and IX (Glossopharyngeal nerve) has been observed in animal models [13,14,15,16,17,18,19,20,21,22,23,24,25,26,27]. In humans, strong evidence that IAV is able to invade the CNS via the olfactory route was shown in a child who was infected with a seasonal H3N2 virus [28]. To our knowledge, there are no convincing data that IAV enters the CNS via the hematogenous route in mammals, which might be due to the fact that this is rarely studied.

**Figure 1:**
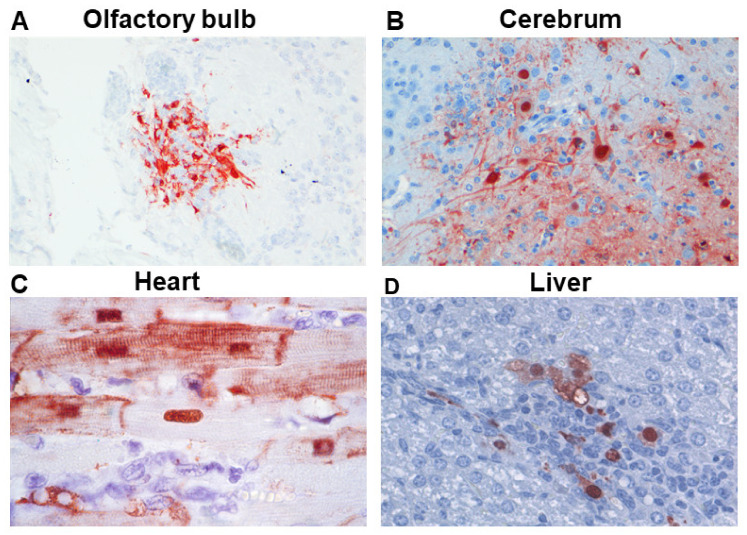
Influenza viral antigen in different organs and animal models. (**A**) HPAI H5N1 virus in the olfactory bulb of intranasal inoculated ferrets. Viral antigen is detected in cells surrounding the glomeruli. (**B**) HPAI H5N1 virus in the cerebrum of an intranasal inoculated ferret. Viral antigen is detected predominantly in neurons (adapted from Schrauwen et al. [15]). (**C**) HPAI H5N1 virus in the heart of a H5N1 intratracheal inoculated cat (adapted from Rimmelzwaan et al., 2006 [47]). Viral antigen is detected in cardiac myocytes. (**D**) 1918 H1N1 virus in the liver of an intranasal inoculated ferret. Viral antigen is detected in hepatocytes (adapted from Figure 5 of de Wit et al. [24], reproduced by permission of Oxford University Press).

Intranasal inoculation of ferrets with different IAVs revealed that there is a large heterogeneity in the ability of IAVs to spread to the CNS [29]. Several studies have shown that i.n. inoculation with HPAIV H5N1 result in virus spread along the olfactory nerve to the CNS resulting in moderate to severe lesions, including meningo-encephalitis, hemorrhages, choroiditis, and ependymitis [13,17,30,31]. Viral antigen could be detected initially in olfactory receptor neurons of the olfactory mucosa, and subsequently, in the glomerular layer of the olfactory bulb, meningeal cells, and later in the neurons of the cerebrum (Figure 1A,B). This was associated with histological lesions in tissue juxtaposed to the CSF in the subarachnoidal space and ventricular system in conjunction with infiltration of mononuclear inflammatory cells [15,29,32]. Intranasal inoculation of HPAIVs H7N7 and H7N9 resulted in the detection of viral antigen in neurons of the olfactory bulb, brain stem, and cerebral cortex [33,34]. Intranasal inoculation of the 1918 H1N1 virus resulted in virus spread via the trigeminal nerve to the brainstem. However, virus spread in the CNS was limited, and without histological lesions [24]. In contrast to zoonotic and pandemic IAV subtypes, detection of seasonal IAV or viral antigen in the CNS is rare [29,31]. Direct comparison of i.n. inoculation and aerosolized virus exposure of HPAIV H5N1 revealed that virus invasion into the CNS was associated with high virus titers and the development of a severe encephalomyelitis, which occurred faster in the aerosolized exposed ferrets using a low virus dose [35]. Comparison of i.n. with i.t. inoculation showed that i.n. inoculation resulted in virus spread to the CNS causing a meningo-encephalitis, while the virus did not spread to the CNS in i.t. inoculated ferrets [32].

Similar to ferrets, i.n. inoculation of IAV in mice may lead to virus spread to the CNS via cranial nerves [19,20,21,22,23,36,37]. Intranasal inoculation with HPAIV H5N1, p2009 H1N1 virus, or neuro-adapted WSN resulted in virus spread via the afferent fibers of the olfactory, vagal, or trigeminal nerve. Associated CNS disease ranged from encephalitis to locomotor disturbances and eventually to coma [14,19,27]. Intranasal inoculation of HPAIVs resulted in the detection of viral antigen in neurons of the brainstem solitary nuclei, and subsequently in the hypoglossal, vagal brainstem nuclei, and periglomerular mitral cells in the olfactory bulb, suggesting that the virus entered the CNS via different cranial nerves [14]. Viral antigen in the CNS was associated with encephalitis and activation of microglia [14,38]. Viruses of the subtype H7N7 and H7N9 but not H7N3 were shown to spread systemically and replicate in the mouse brain [34,39].

In rhesus macaques, cynomolgus macaques, and marmosets, i.n. inoculation of HPAIVs H5N1 infrequently led to the detection of viral RNA in the CNS [40,41,42,43,44,45]. Intratracheal inoculation of IAVs (HPAIV H5N1 and 2009 pH1N1 virus) in cynomolgus macaques and African green monkeys rarely led to signs of CNS disease, even though high virus titers were detected in the respiratory tract [40,41,45]. In general, the ability of zoonotic and pandemic IAV strains to spread to the CNS in NHPs seems limited [44].

In hamsters, red foxes, and cats, i.t. inoculation with HPAIV H5N1 resulted in viral antigen detection within the CNS with associated lesions resulting in encephalitis and meningitis [30,46,47]. Dependent on infectious virus dose, g.i. inoculation of HPAIV H5N1 virus in cats induced CNS lesions, but not as extensively as compared to i.t. inoculations [47,48]. Both i.n. and g.i. inoculations with HPAIV H5N1 virus in hamsters resulted in virus detection in the CNS, but histological CNS lesions were not reported [30]. Foxes fed with HPAIV H5N1 virus infected bird carcasses did not show signs of CNS disease, and virus was not detected within the CNS [46].

In summary, mouse and ferret appear best suited to study CNS disease in influenza, whereas the hamster, red fox, cat, and NHPs are not well characterized and, therefore, do not represent robust experimental animal species to model CNS disease.

## 3. Cardiovascular Disease

IAV infections in humans are associated with cardiovascular disease, including myocarditis, ischemic heart disease, and stroke [1,49,50,51]. In addition, vaccination against influenza virus reduces the risk of developing cardiovascular disease [52,53]. Mammalian animal species in which cardiovascular disease associated with IAV infections may be studied include: laboratory mouse, nonhuman primates, ferret, red fox, and cat. Particularly, inoculation with HPAIVs may cause severe cardiovascular disease. Virus infection and replication in cardiomyocytes (Figure 1C), and rarely in endothelium, are associated with cellular damage and inflammation [45,46,54,55]. Inoculation with seasonal IAVs usually does not result in overt histopathological lesions within the heart [29]. The following paragraphs will focus on the specific animal species used to model human IAV-induced cardiovascular disease.

Intranasal inoculation of mice with an IAV may result in respiratory infection and extra-respiratory spread associated with cardiovascular diseases [56,57,58]. In order to model certain cardiovascular diseases, various mouse strains including specific knock-out mouse strains may be inoculated with different human seasonal and pandemic IAVs, avian IAVs, and mouse-adapted IAVs. The causative role of IAV infection and replication on the pathogenesis of IAV-associated cardiovascular disease was modeled in mice for several acute cardiovascular lesions and diseases (implicated strains of IAV in parenthesis): cardiomyocytic damage (HPAIV H5N1, 2009 pH1N1 virus, mouse adapted H3N2 virus, and PR8/H1N1 virus) [54,56,57,59,60,61,62], endothelial damage (HPAIV H5N1, 2009 pH1N1 virus) [55,62], damage to the myocardial conductive system [60,62], atherosclerosis (H3N2 virus, H1N1 virus) [52,53], thrombosis due to excessive expression of tissue factor (1918 H1N1 virus) [63], thrombosis due to IAV interaction with platelets [64] and increased left ventricular mass, and thickening of the left ventricular wall [61].

Inoculation of IAV in nonhuman primates induced cardiovascular disease associated with infection of cardiac myocytes and endothelial cell infection. Intratracheal spray inoculation with HPAIV H5N1 induced myocarditis that co-localized with viral antigen in common marmosets [45]. Following multisite inoculation of cynomolgus macaques, 1918 H1N1 virus was isolated from the hearts [44] and HPAIV H5N1 infected, besides epithelial cells, few endothelial cells of the lungs [65]. Although HPAIV H5N1 was isolated from cynomolgus macaques’ hearts following i.t. inoculation, neither viral antigen nor myocardial lesions were observed in the heart [66].

In ferrets, IAV spread to the cardiovascular system is reported in several studies [15,29,31,32,67,68]. Although overt cardiovascular disease and myocardial lesions were not evident, both IAV 2009 pH1N1 virus and HPAIV H5N1 could be isolated from ferret hearts during the first few days following i.t. inoculation [29] and i.n. inoculation [31]. Infection of endothelial cells in ferrets has been detected sporadically following HPAIV H5N1 inoculation [29]. Influenza-induced thrombocytopenia has been shown in in ferrets, and was most prominent in HPAIV H5N1-inoculated ferrets compared to ferrets inoculated with H3N2 or 2009 pH1N1 virus [69].

Intratracheal inoculation of red foxes with HPAIV H5N1 resulted in cardiomyocytic necrosis and inflammation associated with virus replication and viral antigen in cardiac myocytes. However, when inoculation was performed by feeding on HPAIV H5N1 infected bird carcasses, the virus was excreted from the pharynx but no systemic spread of the virus was found [46]. Intratracheal inoculation of HPAIV H5N1, or feeding on H5N1 virus infected chicken carcasses of cats, resulted in the detection of influenza viral antigen in cardiac myocytes (Figure 1C) and very occasional endothelial cells of the heart [47]. In contrast, in another study, i.g. inoculation of cats resulted in an overwhelming infection of endothelial cells and, to a lesser extent, cardiac myocytes, epicardial cells, and Purkinje cells [48].

## 4. Other Diseases Involving the Eye, Placenta, Fetus, Lacteal Gland, Liver, Pancreas, Intestinal Tract, and Lymphoid Tissues

In addition to CNS and cardiovascular complications, other extra-respiratory diseases have been observed in humans infected with IAV. These include involvement of the ocular, renal, musculoskeletal, hepatic, and endocrine system as well as complications associated with pregnancy [1]. Here, we will discuss animal models that can be used to study the pathogenesis of ocular disease and complications involving pregnancy, transplacental transmission, and lacteal glands. Renal, musculoskeletal, hepatic, and endocrine system complications have not been studied extensively in animal models, but since all these complications—if associated with virus spread to these tissues—require hematogenous virus spread, they are, therefore, discussed together.

### 4.1. Ocular Disease

Conjunctivitis is the principal ocular complication of IAV infections in humans [70]. It is mainly associated with infections of avian IAV H7 subtypes and, to a lesser extent, with seasonal or pandemic IAVs of the H1N1 subtype [1]. Ocular inoculations of IAV have been performed in mice, ferrets, and cynomolgus macaques. However, none of these models accurately mimic the clinical conjunctivitis observed in humans, but do enable us to identify target cells in the conjunctiva and associated lesions.

In mice, i.o. inoculation after corneal scarification with HPAI and low pathogenic avian influenza (LPAI) H7 viruses and HPAIVs H5N1 resulted in productive infection and replication within the eye or conjunctiva, subsequent spread to the respiratory tract, and, for some of the HPAI H7 viruses systemic spread [39,71]. Oseltamivir treatment could prevent virus replication in the eye and subsequent spread to the respiratory tract after intraocular inoculation with AIV H7 subtypes [72].

In ferrets, i.o. inoculation with AIVs of the H5 and H7 subtype, seasonal IAVs H1N1 and H3N2, as well as IAV 2009 pH1N1, resulted in isolation of the virus from conjunctival washes and respiratory samples. Virus infected cells were observed in the lacrimal glandular epithelium of the conjunctiva and ciliary processes [73]. In cynomolgus macaques, i.o. inoculation of IAV H7N9 resulted in virus replication in epithelial cells within the palpebral conjunctiva [33].

### 4.2. Disease Involving Pregnancy, Placenta, Fetus, and Lacteal Gland

Pregnancy is considered a risk factor for developing severe influenza but may involve extra-respiratory complications for the fetus or child as well, such as stillbirth and low birth weights [74]. Vertical transplacental transmission of HPAIV H5N1 was shown by infection of mice fetuses following i.n. inoculation of gravid dams [68].

Intranasally IAV 2009 pH1N1-inoculated ferret neonates infected their mothers, which developed pneumonia and mastitis. Virus infection and replication within the lacteal glandular epithelium occurred after suckling. In addition, infection of the lacteal glandular epithelium could be induced by direct experimental inoculation of the lacteal glands [75]. Similar i.n. inoculation of nursing mother ferrets led to body weight loss, fever, and mortality of neonates [75].

### 4.3. Hematogenous Spread to Other Organs

Renal, hematologic, musculoskeletal, hepatic, and endocrine system complications are described in humans, but the pathogeneses are poorly understood. Occasionally, the virus has been detected in these extra-respiratory tissues in humans, suggesting that hematogenous spread with subsequent infection of the affected organ system might play a role in the pathogenesis [1]. The ability of IAVs to spread hematogenously in mammals varies largely between the different IAV subtypes. Hematogenous spread is most commonly observed after inoculation with HPAIVs—of which the H5N1 virus is studied most extensively—than with other IAVs [76].

Hematogenous spread of IAVs in mice has been observed following i.n. and g.i. inoculation with HPAIVs, but also following i.n. inoculation with LPAIVs, mouse adapted IAVs, and pandemic IAV [61,77,78,79]. For example, viral RNA and infectious virus could be isolated from the blood, spleen, liver, and kidneys of HPAIV and LPAIV H7N9-inoculated mice [79,80]. Virus spread to extra-respiratory organs by virus isolation or by detection of viral antigen has been observed in the liver, pancreas, kidneys, spleen, and perivisceral fat tissue of HPAIV H5N1-inoculated mice [27,77,81], in the heart of IAV 2009 pH1N1-inoculated mice [57,61], and in the pancreas of mouse adapted IAV H1N1-inoculated mice [78]. By means of immunohistochemistry, viral antigen could be detected in splenic macrophages, cardiomyocytes, hepatocytes, pancreatic endocrine cells of the islets of Langerhans, and peritoneal adipocytes [58,81].

In ferrets, hematogenous spread has been detected following i.n., i.t., aerosol, total respiratory tract, or g.i. inoculations [24,35,82,83,84]. As in mice, hematogenous spread of IAVs in ferrets is mainly observed following inoculation with HPAIVs, but has also been detected after i.n. inoculation with the 1918 H1N1 virus [24]. Several studies showed hematogenous spread in ferrets following i.n., g.i., or total respiratory tract inoculations by detection of infectious HPAIVs H5N1 in the blood, tonsils, spleen, intestinal Peyers patches, liver, and pancreas [30,82,83,85]. Viral antigens could be detected in lymphocytes of the tonsils, intestinal Peyers patches, mesenteric lymph nodes, and in epithelial cells of bile ducts, pancreatic acini, and in hepatocytes [30,83,84,85]. Total respiratory tract inoculation in ferrets induced earlier hematogenous spread of HPAIV H5N1 than following i.n. inoculation [82]. In addition, blood transfusion from HPAIV H5N1-inoculated ferrets transmitted and infected naive recipient ferrets proving infectious viraemia [86]. Intranasal inoculation with IAV 1918 H1N1 led to the detection of infectious virus in the heart, liver, spleen, kidney, and adrenal glands, although the viral antigen was only detected in the liver (Figure 1D) [24].

Additional systemic hematogenous spread of IAVs, in solid organs other than the CNS, has been observed following i.n. and g.i. inoculation of HPAIV H5N1 in cats [48]; in hamsters following i.n. or g.i. inoculations with HPAIV H5N1 [30]; in marmosets following inoculation of HPAIV H5N1 using a tracheal spray [45]; in macaques following i.n. and i.t. inoculations of IAV 1918 H1N1 [44] and following aerosol inoculation with HPAIV H5N1 [40]; and in red foxes i.t-inoculated with HPAIV H5N1 [46].
**Extra-respiratory spread of IAVs in birds** Influenza A virus (IAV) inoculation of birds does not represent an ideal in vivo model to study the extra-respiratory systemic pathogenesis of influenza in humans. There are substantial differences in the pathogenesis of influenza between birds and mammals, and also among different bird species, which compromises comparison. However, this does not imply that studying the pathogenesis of influenza in birds cannot lead to findings that can be extrapolated to the mammalian situation. Intravenous inoculation in six-week-old chickens is still used for the determination of the intravenous pathogenicity index (IVPI) that categorizes the pathogenicity of IAVs. Here, we will in short describe the pathogenesis of IAV infections in birds to highlight the differences with humans and other mammals. Within birds, IAV infection is associated with a different pattern of disease in water birds compared to poultry. Wild ducks and geese are considered the main natural hosts of IAVs and typically do not show signs of disease [87]. Like in humans, epithelial cells are the main target of infection in these birds; however, the virus mainly infects the intestinal epithelium, and less frequently the respiratory epithelium [88]. Poultry, and several wild bird species, can be infected by low pathogenic avian influenza viruses (LPAIVs) and by highly pathogenic avian influenza viruses (HPAIVs) [89]. Infection with LPAIVs in poultry typically produces limited clinical signs, and the virus preferentially targets epithelium of the respiratory tract. Infection with HPAIVs of the H5 and H7 subtypes is associated with severe systemic disease in poultry. HPAIVs target endothelial cells of multiple organs, such as lung, liver, heart, kidney, spleen, pancreas, bursa, and brain. The endotheliotropism of HPAIVs in poultry, which is not observed in humans, is associated with oedema formation and hemorrhages, coagulation disturbances, and induction of acute pro-inflammatory cytokines (cytokine storm), which may lead to acute mortality [90]. In wild birds, HPAIVs predominantly infect epithelial cells of the respiratory tract but not endothelial cells. Infection may lead to infection of parenchymal cells of other organ systems as well, associated with multi-organ necrosis and inflammation [91]. Amongst wild birds, the black swan is an exception because HPAIVs target systemic endothelial cells [92].

## 5. Discussion & Recommendations

**Figure 2:**
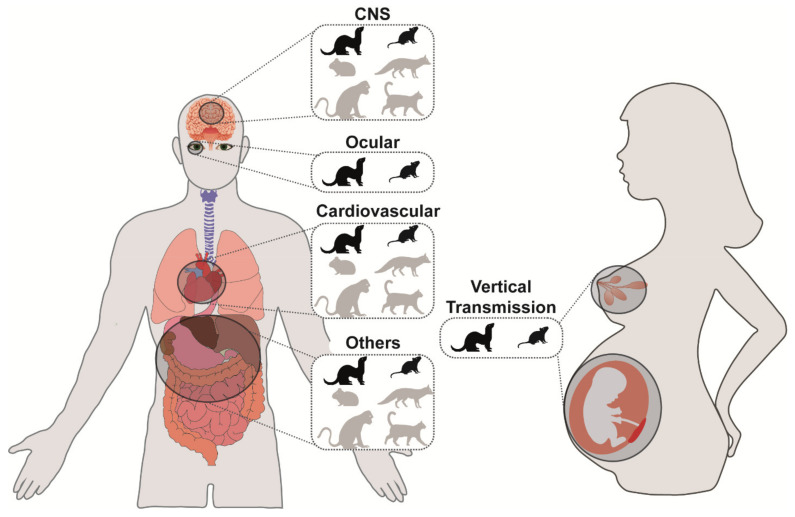
Animal models to study different extra-respiratory complication. Circles depict organs in which influenza A viruses have been detected, and which are discussed in the review. In the zoom out, established experimental animal species to study extra-respiratory spread of each organ are represented. In black are the animal models that have been used and described most extensively.

Animal models for extra-respiratory complications of influenza are an indispensable tool to study its pathogenesis, and some models are more frequently used than others (Figure 2). However, these animal models do not always fully recapitulate the disease observed in humans. There are ways to increase the susceptibility of an experimental animal species for infection with and subsequent virulence after experimental IAV inoculation. However, it is important to realize that this may compromise the extrapolative strength of the model. Examples of ways to enhance virus infectivity and replication in preferred anatomical locations may include, but are not limited to: (1) increase of infectious virus dose of the inoculum, (2) alternative virus inoculation routes, (3) virus adaptation to an experimental animal host, (4) use of permissive knock-out animals, and (5) immunosuppression of the experimental animal.

This review highlights the importance of the inoculation route on the pathogenesis, especially on the pathogenesis of extra-respiratory complications. For example, CNS infection and disease occurred more frequently in ferrets inoculated with HPAIV H5N1 via the i.n. route compared to the i.t. route [32]. Intratracheal inoculation of HPAIV H5N1 in red foxes induced systemic virus spread whereas g.i. inoculation did not induce systemic virus spread [46]. These examples clearly demonstrate that the inoculation route, and possibly the primary site of virus replication in the host, plays an important role in the pathogenesis of extra-respiratory disease caused by IAV infection.

Besides virus subtype or inoculation route, alterations in the host immune response could also have an impact on the pathogenesis of extra-respiratory complications. These can be modulated by certain risk factors for increased disease (e.g., obesity or pregnancy [reviewed by Honce et al. [74]), by medication (e.g., immunosuppression [93]), or by using knock-out animals. It is, therefore, important to consider the impact of the host immune status in the systemic pathogenesis of influenza.

Overall, this review outlines several animal models that may be used as valuable tool to gain insights into the pathogenesis of extra-respiratory spread of IAVs. Such models may be implemented also in efficacy studies of vaccines, antivirals, or other therapeutics, by evaluating protection from extra-respiratory spread and disease following IAV challenge. However, at this stage, the association between clinical disease in humans and the observed findings in in vivo models is sometimes challenging, because the options to study functional changes in the different organ systems of the animal models are limited. In general, mice and ferrets—which are both commonly used for IAV research—appear most appropriate to study systemic IAV spread and associated lesions in extra-respiratory tissues. In particular, the route of inoculation in ferrets appears to be a determining factor in the ability to spread beyond the RT. In conclusion, it is recommended to choose the animal species and route of inoculation that are best suited to address specific research questions concerning extra-respiratory spread and disease. Furthermore, when using animals, it is paramount to retrieve the most accurate and significant data from the different organs of interest in the study. Consequently, in addition to extensive virological analysis, it is recommended to include pathological examination, and where possible, include corresponding functional analysis, as the combined data will provide invaluable insights into the pathogenesis.
